# Prognostic and Immunological Role of Gasdermin E in Pan-Cancer Analysis

**DOI:** 10.3389/fonc.2021.706266

**Published:** 2021-07-26

**Authors:** Zheng Zhang, Shuangshuang Zhao, Haizhen Yang, Yanwei Chen, Huahui Feng, Maohui An, Baoding Chen

**Affiliations:** Department of Medical Ultrasound, Affiliated Hospital of Jiangsu University, Zhenjiang, China

**Keywords:** gasdermin E, pan-cancer analysis, immune infiltrate, survival analysis, pyroptosis

## Abstract

Despite accumulating cell- or animal-based experiments providing the relationship between Gasdermin E (GSDME) and human diseases, especially in malignant cancers, no pan-cancer analysis about the function of GSMDE in cancer management can be available up to date. Our research, for the first time, explored the potential carcinogenic role of GSDME across 33 tumors from the public platform of TCGA (The cancer genome atlas) database. GSDME is highly expressed in most malignant cancers, and obvious relationship exists between GSDME level and survival prognosis of cancer patients. The expression of GSDME was statically associated with the cancer-associated fibroblast infiltration in diverse cancer types, such as BLCA, CHOL, GBM, KIRC, LIHC, MESO, STAD, and UCEC. Furthermore, pyroptosis, sensory perception of sound, and defense response to bacterium were involved in the functional mechanisms of GSDME expression from GO analysis. Last but not the least, *in vitro* experiments were also performed to identify GSDME-induced pyroptosis. Our first pan-cancer analysis of GSDME not only broadens the understanding of the carcinogenic roles of GSDME but also provides a promising therapeutic strategy for benefiting an increasing number of cancerous patients based on GSDME-induced pyroptosis.

## Introduction

As ranking second among human diseases in the United States, malignant cancer has become a major threat to human life worldwide, which has raised great attention of scientists in different fields (1, 2). In view of the sophistication of carcinogenesis, it is extremely urgent to accomplish a pan-cancer analysis of any interested genes and exploit their association with potential molecular mechanisms and underlying clinical prognosis (3). Although great efforts have been devoted in early diagnosis of diverse kinds of cancers and the adoption of innovative approaches to decrease the mortality, the incidence of malignant cancer remains a major concern of human health (4, 5). Fortunately, with the advanced progress of genome-wide sequencing technology, an amount of functional genomics databases can be available from public platforms, such as The Cancer Genome Atlas (TCGA) project (6–9).

The Gasdermin E (GSDME), originally known as deafness, autosomal dominant 5 (DFNA5), was discovered on chromosome 7p15.3 with a peculiar form of autosomal dominant, progressive, sensorineural, and non-syndromic deafness (10). GSDME is one of the Gasdermin (GSDM) families, which possesses its nomenclature to the high-expression pattern along the gastrointestinal tract and skin (dermis) (11–16). It is interesting that GSDME can be cleaved by caspase-3 into pore-formation GSDME-N domain, as a result converting noninflammatory apoptosis to inflammatory pyroptosis in cancer cells (17). On one hand, GSDME is low expression in most cancers and decreased GSDME levels are also related to reduced survival prognosis, indicating that GSDME might function as a role of tumor suppressor (18, 19). However, on other hand, the inflammatory factors released during dysregulation of pyroptosis are widely associated with the tumorigenesis, as well as their drug resistance to chemotherapy (20–22). The function of GSDME in cancer has been increasingly prominent as the investigations has advanced. The structure and function research of GSDME has also been carried out from the aspects of physiology and pathology in various cancers; however, there is still no pan-cancer analysis about association between GSDME and different cancer types from public clinical information.

Our research, for the first time, utilized the TCGA database to perform a pan-cancer expression analysis of GSDME and included a series of relevant studies, including GSDME expression, clinical survival prognosis, immune cell infiltration, and potential signaling pathway, to explore the underlying mechanism in the tumorigenesis and tumor suppression across different cancer species. We strongly believe that the analysis of GSDME could assist in predicting the survival of cancer patients and further deepen our understanding of the individual management for cancer precision therapy.

## Materials And Methods

### Raw Data Acquisition and Processing

We entered DFNA5 into the “Gene_DE” module of TIMER2 website (http://timer.cistrome.org/). Then we scanned DFNA5 expression difference between malignant cancers and corresponding normal tissues for diverse cancers and certain subtypes. To solve the imbalance between the tumor and normal data, which can cause inefficiency in various differential analyses, TCGA and GTEx gene expression data were available from GEPIA (Gene Expression Profiling Interactive Analysis) web server (http://gepia.cancer-pku.cn/#analysis) that are re-computed from raw RNA-Seq data by the UCSC Xena project based on a uniform pipeline, thus allowing for the formation of the most comprehensive expression information (23). Herein, for specific cancers with limited normal and without normal tissues, we applied the “Expression analysis-BoxPlot” module and clicked “Match TCGA normal and GTEx data” module of GEPIA to analyze DFNA5 level between the malignant cancers and adjacent normal tissues.

### Survival Analysis and Relationship With Clinical Stage

The overall survival (OS) and disease-free survival (DFS) information of GSDME within all TCGA cancers were obtained from the “Survival Map” module of GEPIA2. Cutoff-high (50%) and cutoff-low (50%) values were utilized to be thresholds to separate the high- and low-expression cases. In addition, the log-rank test was applied for the hypothesis test, and the survival curves were then graphed *via* the “Survival Analysis” module. Furthermore, the violin images of the GSDME in various pathological stages were also required from the “Pathological Stage Plot” module.

### Genetic Alteration Analysis of GSDME

The genetic alteration features of DFNA5 were queried from the “TCGA Pan Cancer Atlas Studies” module of cBioPortal web (https://www.cbioportal.org/) (24, 25). The information of the alteration frequency, mutation type, and copy number alteration (CNA) were shown in the “Cancer Types Summary” module.

### GSDME-Related Immune Infiltration Analysis

The immune infiltration cells of CD4^+^ T-cells and cancer-associated fibroblasts were chosen for immune infiltration evaluation by through TIMER, CIBERSORT, XCELL, EPIC, QUANTISEQ, MCPCOUNTER, and TIDE algorithms. Correlation (cor) values and P-values were also calculated by purity-adjusted Spearman’s rank correlation test.

### GSDME-Related Gene Enrichment Analysis

The determined GSDME-binding proteins were downloaded from STRING (https://string-db.org/). We picked up the top 100 GSDME-binding genes from TCGA all cancers and adjacent normal tissues *via* “Similar Gene Detection” module. Then the “correlation analysis” module was used for achieving a pairwise gene Pearson correlation analysis of GSDME and these correlated genes. Furthermore, we applied the “Gene_Corr” module to image the heatmap information of these determined genes. Finally, these gene lists were uploaded into DAVID website (database for annotation, visualization, and integrated discovery) to perform the (gene ontology) enrichment and Kyoto Encyclopedia of Genes and Genomes (KEGG) pathway analysis. The results with P <0.05 were considered as statistically significant, providing credibility for the data analysis.

## Results

**Figure 1:**
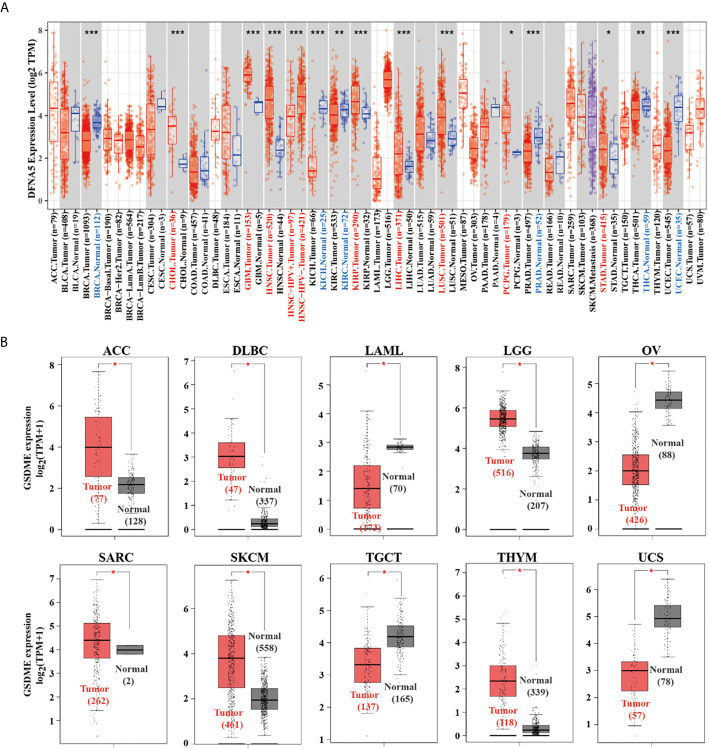
**(A)** The expression difference of the GSDME in diverse cancers or certain cancer subtypes was analyzed *via* TIMER2 algorithm. **(B)** For ACC, DLBC, LAML, LGG, OV, SARC, SKCM, TGCT, THYM, and UCS in the TCGA database, the adjacent normal tissues of the GTEx project were included as controls. The box plots were then graphed. *P < 0.05, **P < 0.01, ***P < 0.001.

As graphed in **Figure 1A**, the expression level of GSDME in malignant cancers of cholangiocarcinoma (CHOL), glioblastoma multiforme (GBM), head and neck squamous cell carcinoma (HNSC), kidney renal papillary cell carcinoma (KIRP), liver hepatocellular carcinoma (LIHC), lung squamous cell carcinoma (LUSC), pheochromocytoma and paraganglioma (PCPG), stomach adenocarcinoma (STAD) is higher than their adjacent normal tissues (P<0.05). After including the normal cases from GTEx project as controls, our group proceeded to perform the expression analysis of GSDME level between the adjacent normal and cancer tissues of adrenocortical carcinoma (ACC), lymphoid neoplasm diffuse large B-cell lymphoma (DLBC), acute myeloid leukemia (LAML), brain lower grade glioma (LGG), ovarian cancer (OV), skin cutaneous melanoma (SKCM), testicular germ cell tumors (TGCT), sarcoma (SARC), thymoma (THYM), and uterine carcinosarcoma (UCS) (**Figure 1B**, P<0.05).

### Clinicopathological Stages and Prognostic Value of GSDME in Cancers

**Figure 2:**
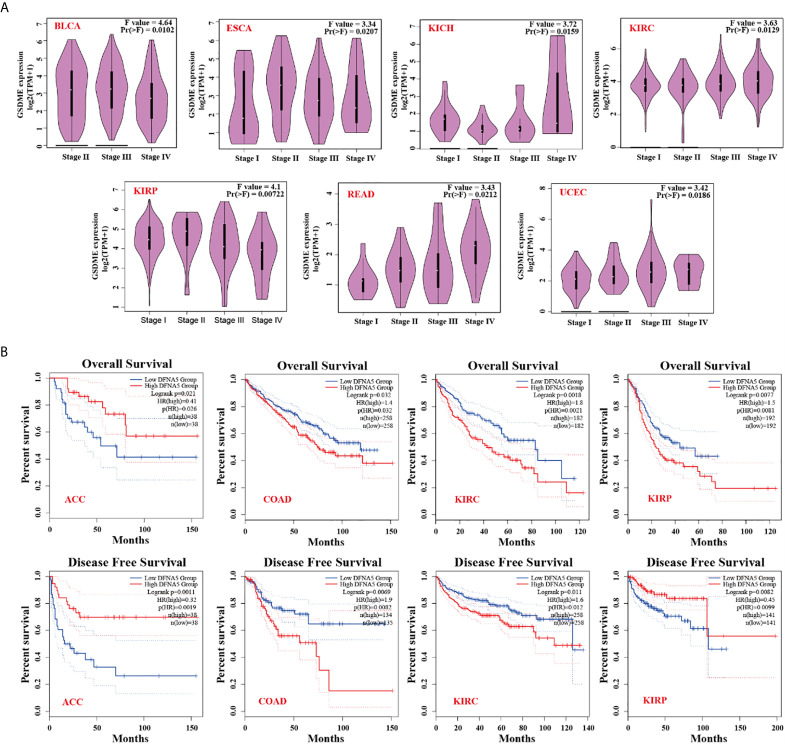
**(A)** Expression status of GSDME in various pathological stages (stage I, stage II, stage III, and stage IV) of BLCA, ESCA, KICH, KIRC, KIRP, READ, and UCEC. **(B)** Correlation of GSMDE expression with overall survival and disease-free survival across different TCGA cancers.

The “Pathological Stage Plot” module of GEPIA was used to analyze the relationship between GSDME expression and the pathological stages of different cancers, including BLCA, ESCA, KICH, KIRC, KIRP, READ, and UCEC (**Figure 2A**, all P value <0.05). We separated the cancer species into high- and low-expression groups, and then explored the association between GSDME level and the clinical prognosis of patients bearing various malignant cancers. As displayed in **Figure 2B**, high expression of GSDME was related to poorer prognosis of overall survival (OS) for malignancies, such as COAD (P=0.032), KIRC (P=0.0021), and KIRP (P=0.0081) across the TCGA database. Disease-free survival (DFS) analysis information revealed an association of high GSDME expression with poorer prognosis of COAD (P=0.0082) and KIRC (P=0.0012). Furthermore, low expression of the GSDME was also associated with poorer OS prognosis for ACC (P=0.026) and DFS prognosis for ACC (P=0.0019) and KIRP (P=0.0099). The abovementioned results suggested that GSDME expression is differentially correlated to the survival prognosis of patients bearing diverse cancers.

### Genetic Alteration Analysis Information

**Figure 3:**
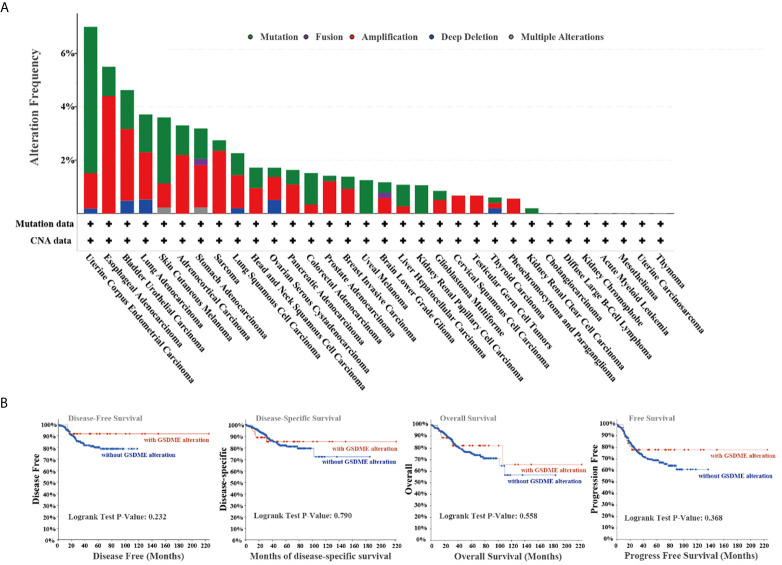
**(A)** Alteration characterizes of GSDME in diverse cancers in TCGA project. **(B)** The potential relationship between mutation status and disease-free, disease-specific, and progression-free survival from the cBioPortal tool.

We studied genetic alteration features of GSDME across the TCGA all cancers. The more than 5.5% mutation frequency of GSDME existed in patients with uterine corpus uterine corpus endometrial cancers. The “amplification” was the dominant type in the esophageal adenocarcinoma types (**Figure 3A**). Moreover, we investigated the possible relationship between the GSDME alteration and relevant survival prognosis. The graphs of **Figure 3B** indicated that CUEC cases with mutated GSDME revealed no association with disease-free survival (P=0.232), disease-specific survival (P=0.790), overall survival (P=0.558), and progression-free survival (P=0.368), as compared with species without GSDME alternation.

### Immune Infiltration Analysis

**Figure 4:**
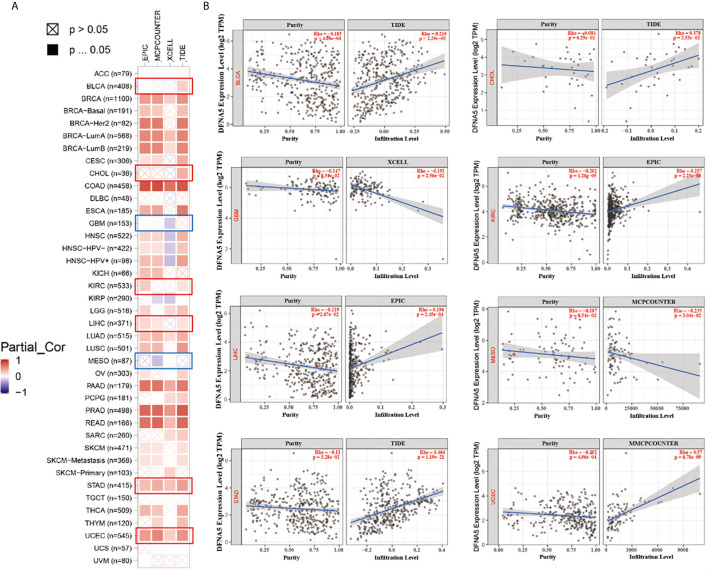
**(A)** The potential correlation between GSDME expression and immune cell infiltration of cancer-associated fibroblasts in all TCGA cancers. **(B)** The scatter plots of related cancers generated by one algorithm, including BLCA, CHOL, GBM, KIRC, LIHC, MESO, STAD and UCEC.

As important components of the tumor microenvironment, tumor-infiltrating immune cells were widely associated with the occurrence, development, or metastasis of malignant cancers (26, 27). Tumor-associated fibroblasts in the tumor microenvironment were shown to take part in regulating the function of different cancer-infiltrating immune cells (28, 29). We applied a series of algorithms to study the potential correlation of GSDME expression with the infiltration level of immune cells in different cancers form TCGA project. We found a positively correlated of GSDME expression with the estimated infiltration value of tumor-associated fibroblasts for BLCA, CHOL, KIRC, LIHC, STAD, and UCEC, and found a negative correlation for GBM and MESO (**Figure 4A**). The scatter plots of these cancers generated by one algorithm are graphed in **Figure 4B**. According to the TIDE algorithm, for instance, the expression of GSDME in BLCA is positively associated with the status of tumor-related fibroblasts infiltration (**Figure 4B**, cor =0.219, P = 2.29e-05).

### Enrichment Analysis and *In Vitro* Experiments

**Figure 5:**
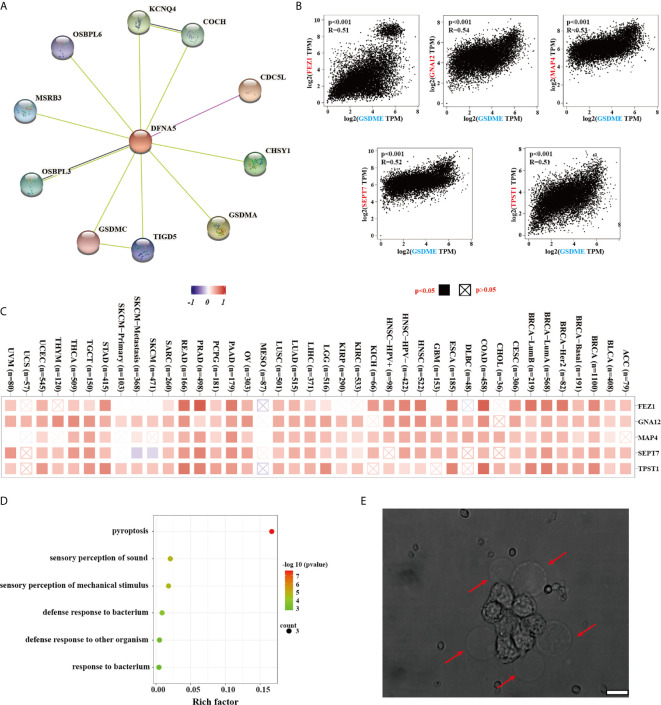
**(A)** The available determined GSDME-related proteins from the STRING website. **(B)** The correlation between GSDME and selected binding genes, such as PEZ1, GNA12, MAP4, SEPT7 TPST1, and TBL2 from the GEPIA2 tool. **(C)** The corresponding heatmap data of different cancer types are graphed in detail. **(D)** GO analysis was displayed based on the GSDME-binding genes. **(E)** The typical morphology of pyroptosis in MCF-7 cells after apoptotic stimuli. Error bar: 200 nm.

To deeply understand the potential mechanism of the GSDME underlying the carcinogenesis or antitumor therapy, our group further made great efforts to pick up the GSDME-related proteins and genes for subsequent enrichment analyses. We acquired the interaction network of 10 GSDME-related proteins, which have been supported by determined evidence (**Figure 5A**). The GSDME expression was statistically related to that of FEZ1 (R=0.51), GNA12 (R=0.54), MAP4 (R=0.53), SEPT7 (R=0.52), and TPST1 (R=0.51) genes (all P values <0.001) (**Figure 5B**). We employed the GEPIA2 tool to require the top 100 GSDME-binding genes (**Figure 5C**). The GO enrichment analysis revealed the majority of binding genes were associated with the cellular biology or pathways of pyroptosis, sensory perception of sound, sensory perception of mechanical stimulus, defense response to bacterium, defense response to other organism, and response to bacterium (**Figure 5D**). Unfortunately, any biological information related to KEGG has been found in our study. Moreover, it is very profound for us to perform *in vitro* experiments to verify GSDME expression for inducing pyroptosis. We used human MCF-7 cells with high expression of GSDME under the stimulation of apoptosis, MCF-7 cancer cells were characterized by cellular swelling, cytoplasmic membrane pore formation, and balloon bulging of membrane rupture (**Figure 5E**). Lactate dehydrogenase (LDH) and adenosine triphosphate (ATP) release are typical characterizes of pyroptotic induction, and we found that the release of LDH and ATP is significantly higher in GSDME expression MCF-7 cells as compared with 4T-1 cells, which can strongly support our points that GSDME expression is a crucial executor for pyroptosis occurrence (**Figures S1**, **2**).

## Discussion

Accumulating evidence has been documented the unique GSDME protein is involved in a series of biological processes in various cancer types, including ontogeny, homeostasis, and pathological processes. Emerging literatures have reported there is a closely relationship between GSDME expression and human diseases, particularly for malignant cancers (30). Unfortunately, the role of GSDME in the cancer pathogenesis and underlying molecular mechanism deserve further investigation. Through previous publications, it is unable to retrieve any available reports about a pan-cancer analysis of GSDME across all tumors. Thus, we carried out a comprehensively screen of GSDME expression, the genetic alteration, immune cell infiltration, and possible signal pathway.

Immunotherapy has completely revolutionized the management of tumor therapy, which restarts and maintains the tumor immune cycle to restore the normal anti-tumor immune activity (31). Unfortunately, currently, immunotherapy only benefits a subset of patients, and one of the reasons is that cancer cells may effectively evade the host immune system caused by inherently immunosuppressive microenvironment (32–36). GSDME is important pore-formation protein for pyroptosis induction, which could be cleaved by caspase-3 into N-terminal fragment of GSDME to squeeze into the lipid contents, form pores in the surface membrane, and drive the cell pyroptosis (37–39). Pyroptosis, an inflammatory programmed cell death (PCD) is triggered by Gasdermin proteins including GSDMA, GSDMB, GSDMC, GSDMD, and GSDME (16, 40–43). Gasdermin proteins have been documented to possess inherent toxic activity of the Gasdermin-N terminal fragments, which are frequently masked by inhibitory Gasdermin-C terminal fragments (19, 44, 45). Proteolytic cleavage the hinge loop between these terminals to unleash the necrotic Gasdermin-N fragments to squeeze the inner leaflet of membrane and form nanopores with a size of ~18 nm in the cellular plasma membrane (44, 46–48). Physical rupture of pyroptotic cells causes the leakage of inflammatory factors IL-18, IL-1β, and DAMPs to amplify the local and systemic inflammatory response, suggesting the immunogenic potential of pyroptosis (49, 50). Pore formation of pyroptosis may lead to the release of immune stimulants, including HMGB1, which can cause dendritic cell (DC) activation and, in turn, propagate antitumor T-cell activity. Moreover, cleaved GSDME can also permeate the mitochondria to positively feedback to the intrinsic apoptotic pathway (16, 51). Thus, therapeutic induction of pyroptosis in tumor cells can be of important value to stimulate a “cold” immunosuppressive microenvironment into “hot” immunogenic microenvironment, thereby eliciting high performance of inhibiting and eliminating malignant tumors (52).

However, despite lots of efforts have made in investigating and integrating information from available TCGA projects, there were still some limitations in the current investigation. First, although bioinformatic analysis offered us some significant insights of GSDME in cancers, it is necessary to conducted biological experiments *in vitro* or *in vivo* for checking on our findings and accelerating clinical application. Further mechanistic explorations will be helpful for clarifying the role of GSDME at the molecular levels. Second, posttranslational modifications are of great value in regulating the activity of intracellular signal transduction and regulatory factors, but post-translational modification data of GSDME is not available in these databases. Third, GSDME expression was found to be associated with cancer immunity and clinical survival prognosis; however, we were not sure that GSDME influenced clinical survival *via* definite signal pathway. Therefore, we are only just beginning to understand the molecular mechanisms of GSDME and its emerging role in cancer research, a great deal of work requires to be done to further broad the applications of GSDME in human cancers.

## Conclusion

In summary, our pan-cancer analysis of GSDME not only broadens understanding of the carcinogenic roles of GSDME, but also provides a promising therapeutic strategy for benefiting an increasing number of cancer patients based on GSDME-induced pyroptosis.

## Data Availability Statement

The original contributions presented in the study are included in the article/**Supplementary Material**, further inquiries can be directed to the corresponding authors.

## Author Contributions

All authors listed have made a substantial, direct, and intellectual contribution to the work and approved it for publication.

## Funding

We greatly acknowledged the Fifth Phase “169 Project” Scientific Research Project of Zhenjiang City (YLJ201931), Zhenjiang Social Development Fund (SH2018035), Zhenjiang Key Research and Development Plan Fund (SH2016036), and the Doctoral Start-up Fund of Affiliated Hospital of Jiangsu University (jdfyRC2019005).

## Conflict of Interest

The authors declare that the research was conducted in the absence of any commercial or financial relationships that could be construed as a potential conflict of interest.

## Publisher’s Note

All claims expressed in this article are solely those of the authors and do not necessarily represent those of their affiliated organizations, or those of the publisher, the editors and the reviewers. Any product that may be evaluated in this article, or claim that may be made by its manufacturer, is not guaranteed or endorsed by the publisher.

## Supplementary Material

Supplementary Figure 1 The release of LDH in MCF-7 and 4T-1 cells under apoptotic stimuli. *P < 0.05; **P < 0.01; ***P < 0.001.

Supplementary Figure 2 The release of ATP in MCF-7 and 4T-1 cells under apoptotic stimuli. *P < 0.05; **P < 0.01; ***P < 0.001
